# Hard carbon microspheres with bimodal size distribution and hierarchical porosity *via* hydrothermal carbonization of trehalose†

**DOI:** 10.1039/d3ra01301d

**Published:** 2023-05-10

**Authors:** Martin Wortmann, Waldemar Keil, Elise Diestelhorst, Michael Westphal, René Haverkamp, Bennet Brockhagen, Jan Biedinger, Laila Bondzio, Christian Weinberger, Dominik Baier, Michael Tiemann, Andreas Hütten, Thomas Hellweg, Günter Reiss, Claudia Schmidt, Klaus Sattler, Natalie Frese

**Affiliations:** a Faculty of Physics, Bielefeld University Universitätsstraße 25 33615 Bielefeld Germany mwortmann@physik.uni-bielefeld.de; b Department of Chemistry, Paderborn University Warburger Straße 100 33098 Paderborn Germany; c Faculty of Engineering and Mathematics, Bielefeld University of Applied Sciences and Arts Interaktion 1 33619 Bielefeld Germany; d Faculty of Chemistry, Physical and Biophysical Chemistry, Bielefeld University Universitätsstraße 25 33615 Bielefeld Germany; e Department of Physics and Astronomy, University of Hawaii Watanabe Hall, 2505 Correa Road Honolulu HI 96822 USA

## Abstract

Hydrothermal carbonization (HTC) is an efficient thermochemical method for the conversion of organic feedstock to carbonaceous solids. HTC of different saccharides is known to produce microspheres (MS) with mostly Gaussian size distribution, which are utilized as functional materials in various applications, both as pristine MS and as a precursor for hard carbon MS. Although the average size of the MS can be influenced by adjusting the process parameters, there is no reliable mechanism to affect their size distribution. Our results demonstrate that HTC of trehalose, in contrast to other saccharides, results in a distinctly bimodal sphere diameter distribution consisting of small spheres with diameters of (2.1 ± 0.2) μm and of large spheres with diameters of (10.4 ± 2.6) μm. Remarkably, after pyrolytic post-carbonization at 1000 °C the MS develop a multimodal pore size distribution with abundant macropores > 100 nm, mesopores > 10 nm and micropores < 2 nm, which were examined by small-angle X-ray scattering and visualized by charge-compensated helium ion microscopy. The bimodal size distribution and hierarchical porosity provide an extraordinary set of properties and potential variables for the tailored synthesis of hierarchical porous carbons, making trehalose-derived hard carbon MS a highly promising material for applications in catalysis, filtration, and energy storage devices.

## Abstract 2

Hydrothermal carbonization of trehalose, in contrast to other saccharides, leads to the formation of microspheres with a bimodal size distribution. The microspheres develop hierarchical porosity with micro-, meso-, and macro-pores after pyrolysis.

## Graphical abstract

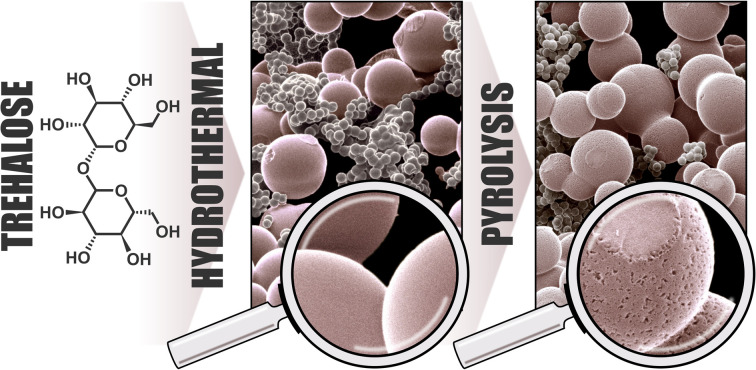

## 1. Introduction

Hydrothermal carbonization (HTC) enables the energy-efficient conversion of sustainable readily-available carbonaceous feedstock into carbon-enriched solids, referred to as hydrochar.^1–3^ Basically any kind of aqueous solution or dispersion of carbonaceous feedstock can be carbonized at low temperatures (usually less than 300 °C) under autogenous pressure. HTC of saccharides is known to produce microspherical hydrochar.^4^ Numerous saccharides, such as glucose,^5,6^ sucrose,^7^ cyclodextrin,^8^ fructose,^9^ xylose,^10^ cellulose,^11^ agarose^12^ and starch,^13^ have been shown to result in microspheres (MS) with mostly Gaussian size distributions. The underlying chemical mechanisms during HTC of saccharides include dehydration, condensation, polymerization, and aromatization.^14,15^ To improve stability, porosity and electrochemical properties, the pristine microspheres (pMS) can be converted into hard carbon microspheres (HCMS), either by high-temperature chemical activation or by purely pyrolytic post-carbonization in an inert atmosphere. The latter has been extensively studied for numerous applications including water treatment,^16–18^ catalyst support materials,^19^ carbon composites,^20–22^ and electrode materials for supercapacitors,^23,24^ fuel cells^25,26^ or batteries.^27–30^ HCMS have the advantage over other forms of hard carbon of high packing density and structural stability with good accessibility of the pores for liquid media such as electrolytes.^31^

**Fig. 1:**
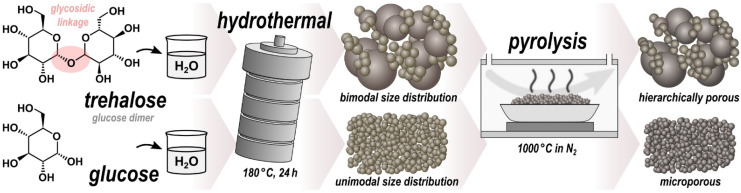
Schematic overview of the synthesis.

In this work, we demonstrate that HTC of trehalose, in contrast to the other saccharides, results in microspherical hydrochar with a distinctly bimodal sphere diameter distribution. Trehalose is a disaccharide consisting of two glucose units bonded *via* a strong α,α-1,1-glycosidic linkage (Fig. 1), making it one of the most chemically stable saccharides.^32^ It is found in numerous organisms, such as bacteria, yeast, fungi, insects, invertebrates, and lower and higher plants, where it primarily but not exclusively serves as a carbon and energy source.^33^ Because of its protective effects against freezing and dehydration, trehalose is already commercially used as ingredient in food, health, and pharmaceutical products, thus being affordable and readily available.^34^ Moreover, trehalose has been found to exhibit bioprotective properties, likely due to its interaction with lipid membranes.^35,36^ Compared to most disaccharides, trehalose is essentially nonreducing, highly resistant to hydrolysis, and more stable to heat and a wide pH range,^32^ all of these strongly affecting the course of chemical reactions involved in HTC.

This study focuses on the morphology, composition, and microstructure of trehalose-derived microspheres (Tre-MS). The composition was examined both in pristine form (Tre-pMS) and after pyrolytic carbonization (Tre-HCMS) using ^13^C solid-state nuclear magnetic resonance spectroscopy (NMR), energy-dispersive X-ray spectroscopy (EDX), X-ray photoelectron spectroscopy (XPS), and Fourier transform infrared spectroscopy (FTIR). The morphology and crystallinity were studied by helium ion microscopy (HIM), nitrogen physisorption, X-ray diffraction (XRD), small-angle X-ray scattering (SAXS) and Raman spectroscopy. The results have been thoroughly compared to glucose-derived microspheres (Glu-MS) before (Glu-pMS) and after pyrolysis (Glu-HCMS). Glucose, the monomer of trehalose, being a more widely used saccharide feedstock for HTC,^37,38^ is particularly suitable for a comparison, as all differences in the resulting MS can be directly attributed to the existence of the glycosidic linkage of trehalose.

Strong compositional similarities were found between Tre-MS and Glu-MS, with a cross-linked furan-based polymeric structure before, and nano-crystalline sp^2^-dominated hard-carbon structure after pyrolysis. However, Tre-MS, in contrast to other microspherical hydrochars, showed a distinctly bimodal size distribution, with both sphere types exhibiting significantly less aggregation and larger sphere diameters than Glu-MS. Remarkably, Tre-MS showed a multimodal pore size distribution at multiple scales with abundant macropores of >50 nm, mesopores of >10 nm and micropores of <2 nm. Such hierarchical porous carbons are highly desirable for a variety of applications as they provide high accessibility of their micropores for a large adsorption capacity both through the voids between the spheres and through macro- and mesopores at the surface of the spheres.^39^ HCMS are widely studied in many fields of material science due to their simple synthesis and favorable properties, however, only few methods are known that can substantially affect the morphology. The bimodal size distribution, large surface area, and hierarchical porosity of Tre-HCMS provide an extraordinary set of properties and potential variables for the tailored synthesis of new hierarchical porous carbons for applications in catalysis, filtration, and especially energy storage devices like super-capacitors or metal ion batteries.

## 2. Materials and methods

### Sample preparation

As illustrated in Fig. 1, the pristine samples Tre-pMS and Glu-pMS were synthesized *via* HTC at 180 °C for 24 h using aqueous solutions of trehalose (≥98% d-(+)-trehalose dihydrate; Carl Roth) and glucose (≥99.5% d-(+)-glucose monohydrate; Carl Roth), respectively. Both solutions were prepared from 2.64 g saccharide (unhydrated mass) and 17.36 g (∼17.4 ml) distilled water. After cooling to room temperature, the pMS were rinsed with distilled water by suction filtration until the water became clear, followed by drying at 60 °C for 12 h. Subsequent pyrolysis was carried out in a tube furnace under a constant nitrogen flow of 150 ml min^−1^ (≥99.999%) at 1000 °C for 1 h (isothermal phase) approached with a constant heating rate of 10 °C min^−1^.

### Characterization

All samples were imaged with a HIM Orion Plus (Carl Zeiss) using an acceleration voltage of 33.4–33.8 kV, a beam current of 0.2–0.4 pA and spot control of 6.5–7.0. In contrast to electron microscopy, scanning with He^+^ ions generates positive surface charges on non-conductive samples, allowing the use of an electron flood gun for charge compensation. The samples could thus be imaged at nanometer resolution without the need for a conductive metal coating.^40^ Experimental parameters were adjusted for each sampling region to optimize charge compensation.

The weight loss during pyrolysis was measured by thermogravimetric analysis (TGA) with a SDT 650 thermoanalyzer (TA Instruments). Differential scanning calorimetry (DSC) was performed with a DSC-3 device (Mettler-Toledo). Both TGA and DSC were measured at 10 °C min^−1^. DSC was measured only up to the device's temperature limit of 500 °C.

The surface area was obtained from nitrogen physisorption experiments carried out with an Autosorb-6B (Quantachrome Corporation) instrument at 77 K; Brunauer–Emmett–Teller (BET) theory was utilized to calculate the BET surface area in a relative pressure range that was determined by Rouquerol criteria.^41,42^

The bulk composition was determined by EDX at 20 kV acceleration voltage using a FEI Helios Nanolab 600 dual beam electron microscope (FEI Germany, now Thermo Fisher Scientific) at three different regions with 2 min acquisition time each (concentration values were averaged).

The surface chemistry was analyzed by XPS in an Omicron Multiprobe Ultra High Vacuum system (Scienta Omicron) at 7 × 10^−11^ mbar using Al K_α_ irradiation. An electron flood gun was used for charge mitigation. Peaks were deconvoluted following a protocol proposed by Smith *et al.*^43^

FTIR spectroscopy in attenuated total reflection (ATR) mode was performed with an FT/IR-4100 spectrometer (JASCO). The spectra were averaged over 32 scans, corrected for CO_2_ and H_2_O absorption and a background correction was applied.

^13^C magic angle spinning (MAS) NMR was performed at a resonance frequency of 75.39 MHz and a temperature of 25 °C using an Apollo spectrometer (Tecmag) equipped with a wide-bore 300 MHz magnet (Oxford Instruments) and a 4 mm MAS probe (Bruker). The spinning frequency was set to 7 or 10 kHz. The spectra were recorded with a spectral width of ±100 kHz, a pulse duration of 3.5 μs, 8192 time-domain data points, 4096 scans, and 5 s recycling delay. An exponential apodization corresponding to a line width of 100 Hz was used to smoothen the spectra. The spectra were externally referenced to the methine carbon signal of adamantane at 29.5 ppm.

SAXS measurements were performed using a Xeuss SAXS/WAXS system (Xenocs) with a Cu K_α_ source (*λ* = 1.541 Å) and a Pilatus 300K hybrid pixel detector (Dectris). The scattering intensity of the sample was normalized with glassy carbon type 2 as a calibration standard.^44^ Samples were measured at three detector distances (2777 cm, 830 cm, 20 cm) to cover a *q*-range from 0.01 Å^−1^ to 2.08 Å^−1^. Only data with a small standard deviation are shown in the overlap regions.

XRD patterns were recorded with a powder diffractometer X'Pert Pro MPD (PANalytical) in Bragg–Brentano configuration using Cu K_α_ radiation.

Raman spectroscopy was carried out with a LabRAM Aramis spectrometer (HORIBA Europe) in backscattering mode with a CCD detector and a 633 nm helium neon laser. A background correction was applied to the spectra.

## 3. Results and discussion

**Fig. 2:**
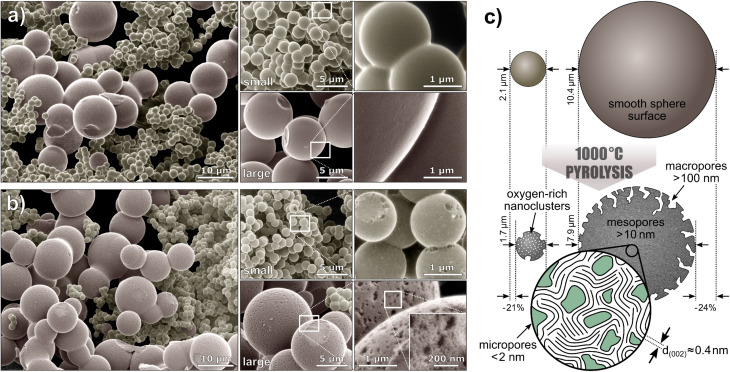
Microsphere morphology of Tre-MS: helium ion microscopy of (a) Tre-pMS before and (b) Tre-HCMS after pyrolysis. The images are colorized and the backgrounds are blackened (unedited images are given in ESI Fig. 3†). The white rectangles indicate magnified image sections. (c) Schematic illustration of the sphere morphology.

**Fig. 3:**
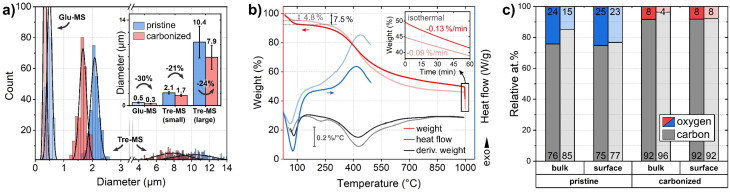
Size and composition of Tre-pMS (blue) and Tre-HCMS (red) compared to Glu-pMS (pale blue) and Glu-HCMS (pale red): (a) sphere diameter distribution (inset shows average values with standard deviation and shrinkage). 6 micrographs were evaluated per sample in different regions (see ESI Fig. 1 and 2†). Each Gaussian represents 250 sphere diameters. The area under the curves does not reflect absolute abundance, as the smaller spheres vastly outnumber the bigger ones. For clarity, the *x*-axis is divided into two areas with different scaling. (b) TGA and DSC measurements during pyrolysis of the pMS. (c) Relative oxygen and carbon content (contents of Glu-MS shown in pale colors) as measured by EDX (bulk: information depth in the micrometer range) and XPS (surface: information depth only few nanometer). The numbers at the bottom and top of the bars represent the carbon and oxygen concentration, respectively.

To examine the sphere morphology, all samples were imaged by HIM before and after pyrolysis (Fig. 2). For accurate sphere diameter determination, multiple sample regions have been imaged;^45^ the results are shown in the ESI Fig. 1 and 2.† The images reveal a polydisperse, distinctly bimodal sphere diameter distribution of Tre-MS (Fig. 3A) with small spheres of (2.1 ± 0.2) μm and large spheres of (10.4 ± 2.6) μm. After pyrolysis, the mean sphere diameters shrank by 21% to (1.7 ± 0.2) μm and by 24% to (7.9 ± 2.0) μm, respectively. Both types are significantly larger and less aggregated compared to Glu-MS (see ESI Fig. 2†). The initially smooth topography of the small and large spheres changed to one exhibiting homogeneously distributed macropores of *ca.* 120–180 nm (notably only at the outer surface, not at breakages) after pyrolysis. However, only the large spheres obtained mesopores of *ca.* 10–20 nm, hardly resolved by HIM. The charge compensation capability of the HIM makes it possible to visualize mesopores in the superstructure of HCMS without conductive coating, which would otherwise be unobservable. As known from studies of sucrose-derived HCMS,^46^ pyrolysis leads to the formation of oxygen-rich nanoclusters between 550 °C and 700 °C, which fully decompose up to 850 °C leaving behind equally sized pores. However, for Tre-HCMS, on the surface of the small spheres (Fig. 2B, top right) such nanoclusters (white spots) can still be seen after pyrolysis at 1000 °C, whereas the clusters on the large spheres have already turned into pores. From this, it can be concluded that the large and small spheres undergo different pyrolytic processes, resulting in differences in composition or density.

The mass decrease during pyrolysis was monitored by TGA (Fig. 3B). Up to about 100 °C, Tre-MS lose about 7.5% moisture by endothermic evaporation, which is substantially more than in the case of Glu-MS, losing only 4.8%. Glu-MS show a small peak in the 1st derivative at 200 °C due to decomposition of residual glucose. No such feature is seen for Tre-MS, indicating precursor-free char formation. Both samples undergo a fast pyrolytic carbonization phase in the same temperature range from *ca.* 300 °C to 500 °C. However, Tre-MS decompose at a lower rate and generate less reaction heat, as measured by DSC. Above 500 °C Tre-MS show a delayed decomposition associated with a higher decomposition rate up to 1000 °C. During the isothermal phase (inset of Fig. 3B), both Tre-MS and Glu-MS show an almost linear mass loss over time, implying that their oxygen content can be precisely controlled. Overall, Tre-MS lost 51.1% and Glu-MS lost 56.3% of their dry mass during pyrolysis. Notably, with 24 at% Tre-pMS had a substantially higher oxygen content compared to Glu-pMS having only 15 at% (Fig. 3C), suggesting that both trehalose and its hydrochar exhibit greater resistance to thermal decomposition.

In Tre-MS no significant difference was observed between surface and bulk oxygen content (Fig. 3C), as measured by XPS and EDX, respectively. The bulk oxygen content was about twice as high in Tre-MS as in Glu-MS, both before and after pyrolysis, which is consistent with the TGA results. Glu-MS showed almost twice as much oxygen at the surface as in the bulk. It should be noted that the accuracy of quantitative EDX measurements on light elements such as carbon and oxygen is controversial, which is why the results should be read with caution.

The chemical structure of saccharide-derived pMS typically consists of a complex network of cross-linked furan rings with some amount of embedded formic and levulinic acid resulting from various reaction routes during HTC involving hydroxymethylfurfural, the dehydrogenation product of glucose.^15^ Chemical information concerning the samples of this study has been obtained by IR, NMR and XPS.

**Fig. 4:**
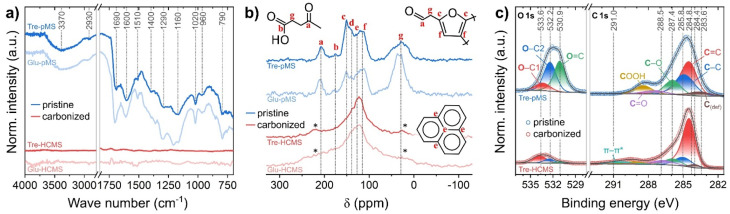
Chemical composition of Tre-pMS (blue) and Tre-HCMS (red) compared to Glu-pMS (pale blue) and Glu-HCMS (pale red): (a) FTIR spectra, (b) ^13^C CP-NMR spectra, (c) deconvoluted XPS core-level spectra (see ESI Fig. 4† for spectra of Glu-MS).

The FTIR spectra of both Tre-pMS and Glu-pMS (Fig. 4A) show the characteristic peaks of saccharide-derived pMS. The presence of aromatic rings is indicated by C=C ring stretch vibrations (1600 cm^−1^; 1510 cm^−1^) and the C–H out-of-plane ring bending vibration (780 cm^−1^). Aliphatic CH_2_ groups cause symmetric and asymmetric stretching and deformation vibrations (2930 cm^−1^; 2860 cm^−1^; 1360 cm^−1^, respectively). The peak at 1690 cm^−1^ is caused by C=O stretching vibrations. The presence of residual water and acids is indicated by the O–H stretching vibration (3000 cm^−1^), O–H deformation vibrations (1400 cm^−1^; 1290 cm^−1^) as well as the C–O stretching vibration (1160 cm^−1^; 1020 cm^−1^). The strong C–O–C stretching vibration band (1160 cm^−1^) is characteristic for furanic ring structures. The spectra of the HCMS exhibit no significant features due to the absence of functional groups in of the oxygen deficient hard carbon structure.

The ^13^C NMR spectra (Fig. 4B) were obtained using cross polarization (CP) for the pMS samples and direct excitation (DE) for the HCMS samples. No ^13^C signals polarized *via*^1^H spins could be acquired for the HCMS confirming scarcity of protons in the samples. In good agreement with reported spectra, the following peak assignments were made based the work by Bacille *et al.*^15^ (the chemical shifts given below correspond to the dotted lines in Fig. 4B): (i) the region above 160 ppm results from carbonyl signals in ketones and aldehydes (a: 200–220 ppm) and carboxylic acid moieties (b: 175 ppm). (ii) The region from 100–160 ppm contains signals from sp^2^–carbon sites, including those from carbon in graphene-like sheets (e: 130 ppm) and carbon in furan ring structures with signals from the 2nd and 5th (c: 151 ppm, d: 140) as well as 3rd and 4th ring position (f: 114 ppm). (iii) The region below 70 ppm (g) corresponds to sp^3^–carbon sites containing signals from mobile and rigid CH_*x*_ (*x* = 1–3) groups. Mind that the assignments shown in Fig. 4B illustrate only the most abundant structures and ignore a broad range of possible structural variations. As Tre-pMS and Glu-pMS are synthesized from very similar precursors, their overall structure, which can be deduced from FTIR and NMR, differs only quantitatively. The aliphatic region below 70 ppm is much less pronounced in Tre-pMS, indicating a lower degree of furan ring openings during HTC, which can be attributed to the high hydrolysis resistance of trehalose. Graphene-like structures are only a minor component of pMS, but dominate the spectra of HCMS. The ε peak of Tre-HCMS is narrower and shifted up-field compared to Glu-HCMS, which is consistent with a lower degree of carbonization (in agreement with TGA and EDX), as the growth of graphitic domains increases the anisotropy of the magnetic susceptibility.^47^

The surface chemistry of the Tre-MS was analyzed and compared to Glu-MS using XPS (Fig. 4C). The C 1s and O 1s spectra of Tre-pMS indicate a typical hydrophilic oxygen-rich char composition with mostly double-bonded oxygen (O=C) and single-bonded oxygen moieties at aliphatic carbon sites (O–C2), and a carbon sp^2^ to sp^3^ ratio of about 1.6. After pyrolysis, the oxygen content of Tre-HCMS has strongly reduced and the majority of the remaining oxygen moieties is now single-bonded to aromatic carbon sites (O–C1). The carbon sp^2^ : sp^3^ ratio increased to about 6.3. The aromatization gives rise to the π–π* shake-up peak, indicating electrical conductivity.^48^ The absolute oxygen content of the sphere surface is 25.2% before and 8.2% after pyrolysis (Fig. 3C), only marginally higher than that of Glu-MS with 23.2% before and 7.8% after pyrolysis.

**Fig. 5:**
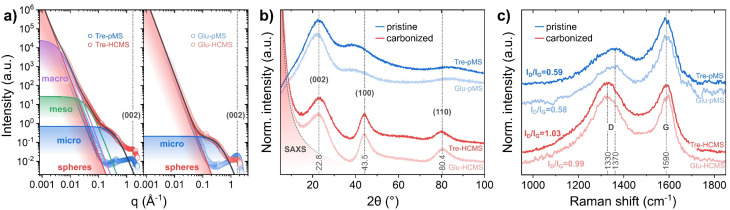
Microstructure of Tre-pMS (blue) and Tre-HCMS (red) compared to Glu-pMS (pale blue) and Glu-HCMS (pale red): (a) deconvoluted SAXS data, (b) XRD pattern, and (c) Raman spectra.

The sphere microstructure has been examined by nitrogen adsorption, SAXS, XRD and Raman spectroscopy (Fig. 5). The pore formation results in a strong increase of the BET surface area from less than 20 m^2^ g^−1^ before pyrolysis to 620 m^2^ g^−1^ for Tre-HCMS and 590 m^2^ g^−1^ for Glu-HCMS (isotherms are shown in ESI Fig. 5†), which is comparable with reported values.^46^ Such values are expected, as the BET surface area is primarily attributable to microporosity. Both carbonizes samples exhibit a type I isotherm shape typical for microporous materials. The uptake of N_2_ above a relative pressure of 0.2, suggests a mesoporous component with a very broad distribution, which cannot be definitively deduced from the isotherms. Here we used SAXS measurements (Fig. 5A) analyzed by the Porod method as described by Stevens and Dahn^49^ (further description in the ESI†) to confirm the microscopic observations: the underlying linear function (red line) corresponds to Porod's law and can be attributed to scattering by inter-spherical voids, which is not substantially affected by pyrolysis and causes the data of pMS and HCMS to overlap at low values of the scattering vector *q*. The Porod exponent *p* in *q*^−*p*^, defining the slope of the double logarithmic plot, was equal to 4 for all samples (the slopes of the pMS and HCMS are congruent), which is consistent with a macroscopically smooth surface. The contribution of different pore sizes extends to different *q* values, with smaller pores contributing at higher *q* values.^50^ Both HCMS samples developed pronounced microporosity after pyrolysis with identical average pore diameters of 1.45 nm, which is characteristic for the hard carbon structure of saccharide-derived HCMS. However, only Tre-HCMS showed significant signal contributions towards lower *q* values attributed to aggregated meso- and macropores with average diameters of 15.2 and 142 nm, respectively, which is in excellent agreement with the HIM images. These results confirm a complete pore hierarchy including inter-spherical porosity as well as macro-, meso- and microporosity in Tre-HCMS.

At higher *q* values, the diffraction peak near *q* = 8 nm^−1^ is attributed to the (002) plane of the graphitic carbon lattice and marks the transition to the XRD data (Fig. 5B). The XRD data show 3 main peaks: those at 43.5° and 80.4° are assigned to the (100) and (110) planes of the graphitic lattice, respectively, and show substantial narrowing due to pyrolysis. The peak at 22.8° assigned to the (002) plane, however, does not change, indicating purely lateral growth of the graphitic domains, typical for a turbostratic hard carbon structure.^46^ The Raman spectra (Fig. 5C) show the D band, which is attributed to disorder in the graphitic lattice and at domain boundaries, and the G band, which is attributed to in-plane sp^2^ ring stretching vibrations. The spectra display the expected increase in the *I*_D_/*I*_G_ height-ratio (from 0.54 to 1.03) and downshift of the D band (from 1370 to 1330 cm^−1^) associated with increasing crystallinity during pyrolysis.^51,52^ Both XRD and Raman spectra indicate a slightly reduced crystallinity of Tre-pMS compared to Glu-pMS and of Tre-HCMS compared to Glu-HCMS, which can be attributed to its higher oxygen content.

As shown above, Tre-HCMS, unlike Glu-HCMS, show a bimodal size distribution and a multimodal pore-size distribution. The origin of the bimodal sphere size distribution has not yet been conclusively elucidated. However, earlier studies have shown that char formation in HTC of saccharides is a discontinuous process that starts abruptly when a critical pH of the process water is reached.^53,54^ Compared to other common saccharide precursors for HTC, trehalose is non-reducing (its anomeric carbons are linked, so that the rings cannot open to form aldehyde groups) and has a high thermal and hydrolytic stability in solution, which could lead to two distinct time-shifted chemical reactions, with an earlier onset of char formation producing larger spheres and a later onset producing smaller spheres. The reason could be that a glucose molecule and a glucose cation are formed upon cleavage of the glycosidic linkage, with the cation being more reactive than the glucose molecule. This could be examined, for example, by a time-dependent investigation of the process water. Reportedly, trehalose possesses a high propensity to form hydrogen bonds with water and other trehalose molecules in aqueous solutions, promoting the concentration-dependent aggregation of trehalose clusters of various sizes,^55^ which might affect its reactivity during HTC. Different starting times for the hydrolysis reaction depending on the state of aggregation would be conceivable. Detailed parameter studies of the HTC process could reveal opportunities to manipulate the resulting size distribution, *e.g.*, by using mixtures of different precursors.

With regard to the porosity, it is yet unclear why different types of spheres developed different kinds of pores. While all spheres developed microporosity typical for hard carbons, only Tre-HCMS, both small and large, showed macropores of comparable size. However, the small spheres had not yet undergone the transition from oxygen-rich nanoclusters to mesopores. The HIM images suggest that meso- and macropores are formed exclusively at the sphere surface of Tre-HCMS, which might be explained by stronger aggregation of oxygen-rich species during pyrolysis due to their bigger diameters in comparison to Glu-HCMS. A similar formation mechanism of meso- and macroporosity was previously observed in sucrose-based HCMS with an intermediate size distribution compared to Tre-HCMS and Glu-HCMS.^45^ Further temperature-resolved pyrolysis studies could elucidate the role of sphere size and composition in the pore formation process and thus shed light on the formation of hierarchical porosity in hard carbons in general.

## 4. Conclusion

It was demonstrated that microspherical hydrochar with distinctly bimodal sphere diameter distribution and hierarchical porosity can be synthesized by single-step HTC of trehalose. Although physicochemical properties, such as chemical structure, crystallinity and microporosity, are similar to glucose-derived MS, a substantially higher oxygen content was observed both before and after subsequent pyrolysis at 1000 °C, which can be attributed to the temperature and hydrolysis resistance of trehalose. HIM imaging has shown that pore formation in the small spheres is not yet complete at 1000 °C, suggesting that their porosity can be tuned independently from the large spheres.

After pyrolysis, Tre-HCMS, in contrast to other saccharide-derived HCMS, provide not only the typical inter-spherical porosity with microporosity of the hard carbon structure, but also macro- and mesoporosity at the sphere surface. Hierarchical porous carbons, especially those derived from bio-mass, have attracted considerable interest for the synergistic effect of macro-, meso-, and microporosity in applications such as catalysts, drug delivery, and especially energy storage, where such properties have already proven beneficial.^56–59^ Moreover, in such fields, a bimodal sphere diameter distribution could facilitate parallel interactions with the surrounding medium like catalysis of different chemical reactions, time-shifted drug release or optimized energy storage. Tre-HCMS are particularly promising due to their simple and energy-efficient synthesis as well as the sustainability and availability of trehalose. Their performance in practical applications is subject to ongoing research.

## Author contributions

W. K. and C. S. performed/evaluated NMR, E. D. and K. S. prepared samples by HTC, M. We. and N. F. made HIM images and XPS measurements, R. H. and T. H. performed/evaluated SAXS, B. B. prepared samples by pyrolysis, J. B. and G. R. performed/evaluated XRD, L. B. and A. H. performed/evaluated TGA measurements, C. W., D. B., and M. T. performed/evaluated N_2_ adsorption measurements, M. Wo., K. S., and N. F. planned and supervised the experimental work, M. Wo. wrote most of the text with the assistance of all co-authors, M. T., A. H., T. H., G. R., C. S., and N. F. revised the manuscript.

## Conflicts of interest

The authors declare no competing interests.

## Supplementary Material

RA-013-D3RA01301D-s001
