# Supplementary material for: Hard carbon microspheres with bimodal size distribution and hierarchical porosity via hydrothermal carbonization of trehalose
Source: RSC Adv. 2023 May 10;13(21):14181–9. doi: 10.1039/d3ra01301d (PMC10170240; doi:10.1039/d3ra01301d)
Supplement: RA-013-D3RA01301D-s001 [file RA-013-D3RA01301D-s001.pdf]

Electronic Supplementary Material (ESI) for RSC Advances.
This journal is © The Royal Society of Chemistry 2023

Supplementary Material:

Hard Carbon Microspheres with Bimodal Size Distribution and Hierarchical Porosity via Hydrothermal Carbonization of Trehalose

Martin Wortmann [a,*], Waldemar Keil [b], Elise Diestelhorst [c], Michael Westphal [a], René Haverkamp [d], Bennet Brockhagen [c], Jan Biedinger [a], Laila Bondzio [a], Christian Weinberger [b], Dominik Baier [b], Michael Tiemann [b], Andreas Hütten [a], Thomas Hellweg [d], Günter Reiss [a], Claudia Schmidt [b], Klaus Sattler [e], Natalie Frese [a,e]

[a] Bielefeld University, Faculty of Physics, Universitätsstraße 25, 33615 Bielefeld, Germany
[b] Paderborn University, Department of Chemistry, Warburger Straße 100, 33098 Paderborn, Germany
[c] Bielefeld University of Applied Sciences, Faculty of Engineering and Mathematics, Interaktion 1, 33619 Bielefeld, Germany
[d] Bielefeld University, Faculty of Chemistry, Physical and Biophysical Chemistry, Universitätsstraße 25, 33615 Bielefeld, Germany
[e] University of Hawaii, Department of Physics and Astronomy, Watanabe Hall, 2505 Correa Road, Honolulu, HI 96822, USA

***** Correspondence: mwortmann@physik.uni-bielefeld.de

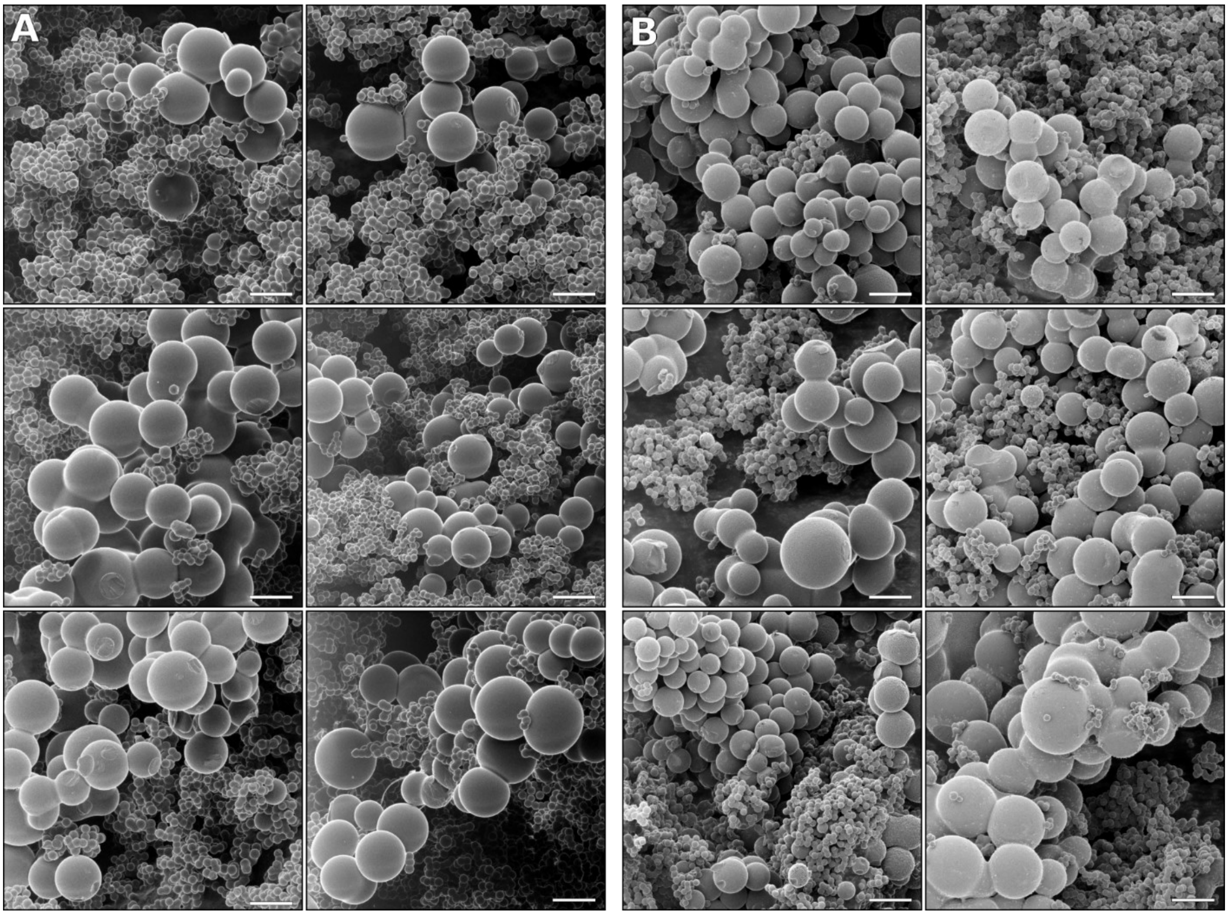

Supplementary Figure 1: HIM images of (**A**) Tre-pMS before and (**B**) Tre-HCMS after pyrolysis. Scale bars are 10 µm.

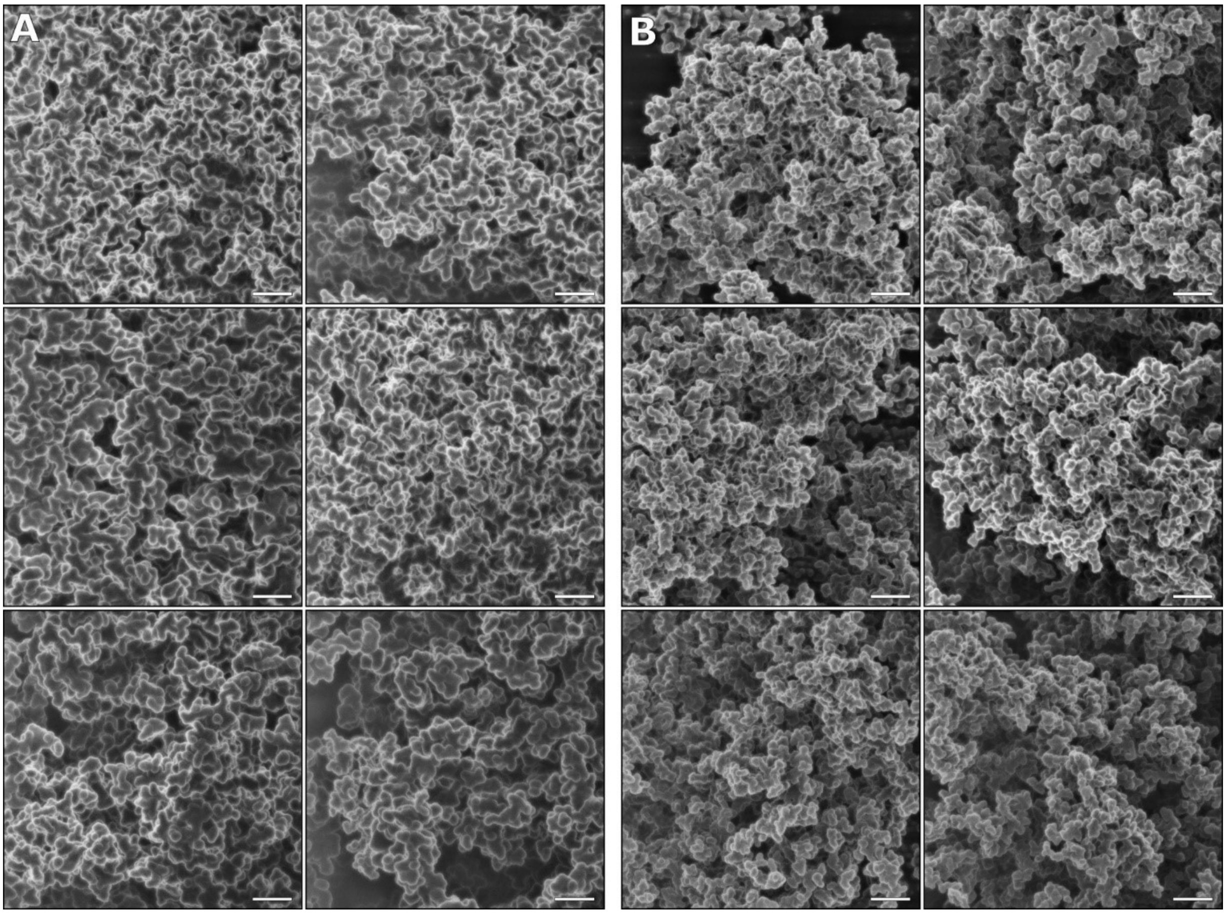

Supplementary Figure 2: HIM images of (**A**) Glu-pMS before and (**B**) Glu-HCMS after pyrolysis. Scale bars are 2 µm.

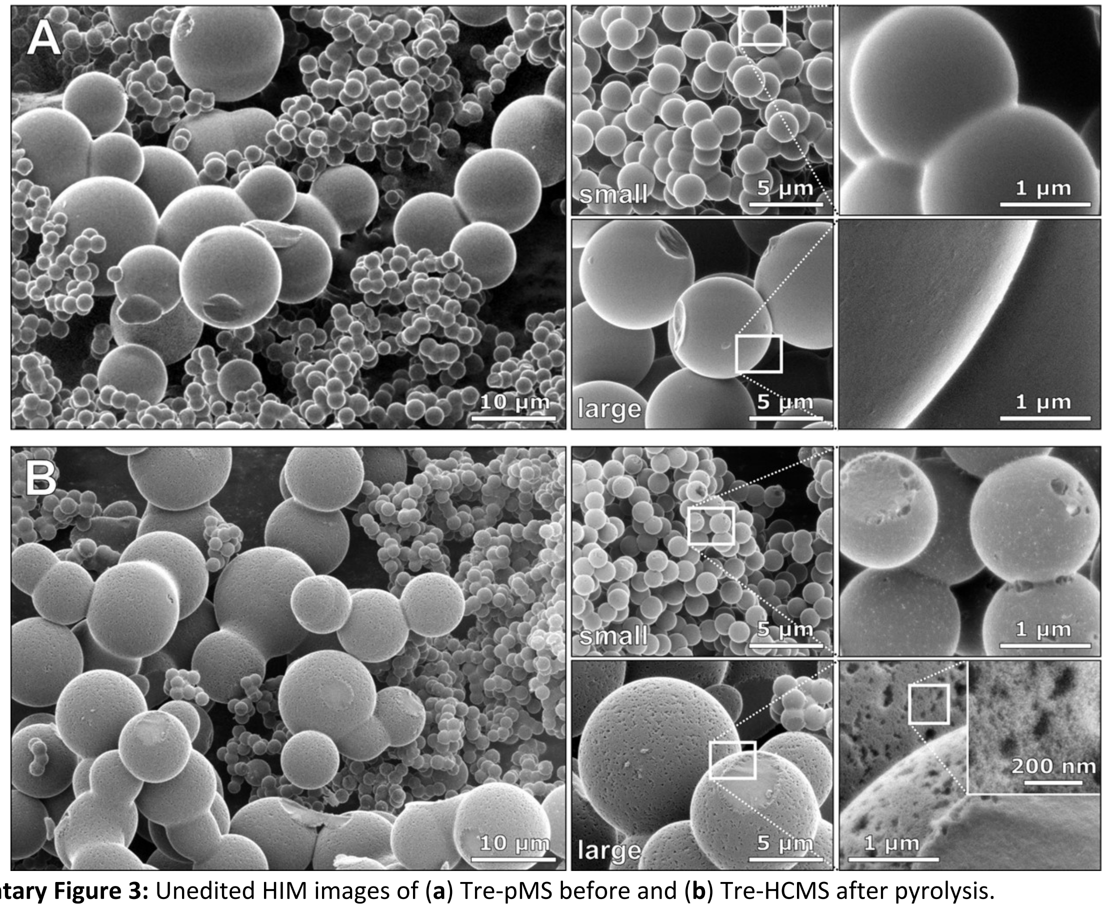

Supplementary Figure 3: Unedited HIM images of (**a**) Tre-pMS before and (**b**) Tre-HCMS after pyrolysis.

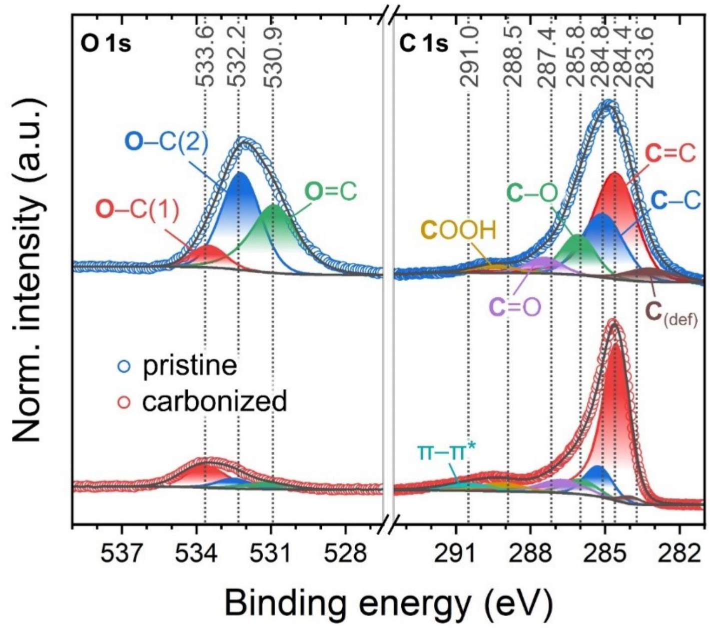

Supplementary Figure 4: XPS core-level spectra of Glu-pMS and Glu-HCMS.

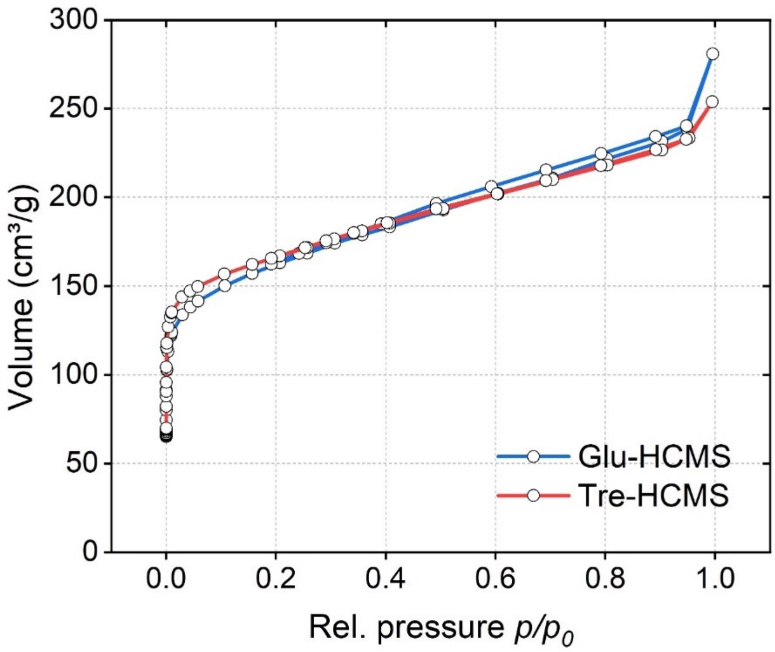

Supplementary Figure 5: Nitrogen adsorption isotherms of Tre-HCMS and Glu-HCMS.

Supplementary Information on SAXS Measurements:

The SAXS data (Figure 4in the paper) were deconvoluted according to the Debye-Bueche model [1,2]:

$$I(Q) = I_{porod} + I_{pores} \tag{1}$$

$$I_{porod} = \frac{A}{Q^p} \tag{2}$$

$$I_{pores} = \sum_{i=1}^{N} \frac{B_i}{(1 + \xi_i^2 \cdot Q^2)^2} \quad (3)$$

I_{porod} and I_{pores} are the signal contributions of the macroscopic sphere surfaces and the pores within the spheres, respectively. Here, the sum over *N* pore sizes is taken to account for a multimodal pore size distribution. *A* and B_i are constants related to the total surface area and the scattering length density contrast of the sample. *p* is the Porod exponent, which is related to the smoothness of the sphere surface. ξ_i is the characteristic length over which electron density variations occur, related to the radius of gyration of the scattering object, i.e. the pores [3].

The scattering vector *q* is defined by

$$q = \frac{4\pi}{\lambda} \sin\left(\frac{\theta}{2}\right) \quad (4)$$

where λ is the wavelength and θ is the scattering angle.

The average pore diameter *d* is given by

$$d = 2 \cdot \xi_i \cdot \sqrt{10}.$$

The fit parameters and the resulting correlation lengths are listed in Supplementary Table 1. Three different pore sizes were found for Tre-HCMS, resulting in two additional parameters B_2 and B_3.

Supplementary Table 1: Fit parameters for the data shown in Figure 4.

sample	A (Å^{-p})	p	B_1	ξ_1 (Å)	d_1 (nm)	B_2	ξ_2 (Å)	d_2 (nm)	B_3	ξ_3 (Å)	d_3 (nm)
Tre-HCMS	2.3e-06	4	0.70	2.3	1.45	26.54	24	15.18	25629	225	142.30
Glu-HCMS	3.5e-06	4	0.21	2.3	1.45	-	-		-	-	

Supplementary References

[1] Debye, P., Anderson Jr, H. R., & Brumberger, H. (**1957**). Scattering by an inhomogeneous solid. II. The correlation function and its application. Journal of applied Physics, 28(6), 679-683. DOI: 10.1063/1.1722830

[2] Debye, P., & Bueche, A. M. (**1949**). Scattering by an inhomogeneous solid. Journal of Applied Physics, 20(6), 518-525. DOI: 10.1063/1.1698419

[3] Stevens, D. A., & Dahn, J. R. (**2000**). An in situ small-angle X-ray scattering study of sodium insertion into a nanoporous carbon anode material within an operating electrochemical cell. Journal of The Electrochemical Society, 147(12), 4428. DOI: 10.1149/1.1394081
